# Estimating the potential health effects of cleaner air in the initial stages of the COVID-19 pandemic: a study in Malmö, Sweden

**DOI:** 10.1080/16549716.2024.2416291

**Published:** 2024-10-22

**Authors:** Ebba Malmqvist, Emilie Stroh, Erin Flanagan, Christina Isaxon, Pontus Roldin, Anna Oudin

**Affiliations:** a Division for Occupational and Environmental Medicine, Lund University, Lund, Sweden; b Ergonomics and Aerosol Technology, Lund University, Lund, Sweden; c IVL, Swedish Environmental Research Institute, Malmö, Sweden; d Department of Physics, Lund University, Lund, Sweden; e Sustainable Health, Umeå University, Umeå, Sweden

**Keywords:** Health impact assessment, air pollution, air quality, environmental health, urban health, transportation, place and health

## Abstract

**Background:**

During the initial phase of the COVID-19 pandemic, reductions in air pollution were globally observed owing to decreased human activities, underscoring the potential for cleaner air through shifts in human behaviour.

**Objectives:**

The objective of the present study was to hypothetically estimate the resulting population health impacts in Malmö, Sweden, if these improvements in air quality were to become permanent.

**Methods:**

We utilized air pollution data from two measurement campaigns conducted in the spring of 2019 and the spring of 2020 for our Health Impact Assessment, applying standard methods. This assessment involved making assumptions about baseline population risk and using established concentration-response functions.

**Results:**

In the spring of 2020, the NO2 concentrations exhibited an average decrease of 6.6 μg/m3 (42%) decrease and PM2.5 concentrations a 1.9 μg/m3 (22%) decrease, compared to the spring of 2019. If sustained, such improvements could lead to an estimated 1–3% decrease in premature deaths, a 2% decrease in preeclampsia cases, a 6% decrease in low birthweight children, a 4% decrease in bronchitis cases among children, a 2% decrease in asthma cases, a 0.2% decrease in hospital admissions for respiratory diagnoses, and an estimated 11% decrease in dementia cases annually.

**Conclusion:**

The findings illustrate the potential for enhanced health in Malmö due to improved air quality. Efforts to combat air pollution and implement long-term strategies, such as those targeting urban mobility and commuting patterns, are essential for the health and well-being of both local and global populations.

## Paper context

- **Main findings:** Air quality improvements, akin to those seen during the early COVID-19 phase, could prevent 1–3% of premature deaths, benefiting population health.
- **Added knowledge:** The study emphasizes human activities’ impact on local air pollution, and the potential for health protection through pollution reduction.
- **Global health impact for policy and action:** Such findings stress the urgency for enduring solutions to combat air pollution.

## Background

In March 2020, drastic measures were adopted around the globe to limit the spread of the novel SARS-CoV-2 virus when the World Health Organization (WHO) declared COVID-19 a pandemic. The pandemic has rapidly led to some of the most pervasive changes in economic activity around the world. While the duration and extent of these adaptations were not permanent, they had a substantial impact on transportation and commuting patterns, and consequently, on air pollution levels across the globe [1]. Ambient air pollution exposure is one of the major causes of morbidity and mortality worldwide, which has led the WHO to lower its Air Quality Guidelines substantially in 2021 [2]. The primary aim of the current study was to estimate the potential population health impacts if such changes in air pollution concentrations were permanent.

## Material and methods

### Study setting

The present study was performed in Malmö, Sweden, a country which, contrary to many others, did not enforce formal lockdowns but instead encouraged citizens to work from home when possible, temporarily recommended secondary and post-secondary education to be held remotely, and suggested limiting non-essential travel, both within the country and abroad. This led to changes in mobility patterns which, influencing air pollution concentrations. The total population of Malmö for the year 2016 (*N* = 326,092) was used for the calculations. Table 1 outlines the study population for each included health outcome.

**Table 1.** Population, relative risk (RR) and 95% confidence interval (95% CI) assumptions applied in the health impact assessment.

Health outcome	Age	RR (95% CI)	Source
Mortality			
	≥30 years	1.06 (1.03–1.08) per 10 μg/m^3^ increase of NO_2_	Héroux et al. [3]
		1.08 (1.01–1.14) per 10 μg/m^3^ increase of NO_2_	Raaschou-Nielsen et al. [4]
		1.02 (1.01–1.04) per 10 μg/m^3^ increase of NO_x_	Stockfelt et al. [5]
		1.06 (1.04–1.08) per 10 μg/m^3^ increase of total PM_2.5_	Héroux et al. [3]
		1.26 (1.19–1.34) per 10 μg/m^3^ increase of local PM_2.5_	Turner et al. [6]
Morbidity			
Preeclampsia	Women 15–44 years	1.13 (1.07–1.19) per 10 μg/m^3^ increase of NO_x_	Malmqvist et al. [7]
Low birth weight*	Births among women 15–44 years	1.09 (1.03–1.15) per 10 μg/m^3^ increase of PM_2.5_	Perera et al. [8]
Bronchitis	5–14 years	1.02 (0.99–1.06) per 1 μg/m^3^ increase of NO_2_	McConnell et al. [9]
Asthma incidence	5–14 years	1.05 (1.02–1.07) per 4 μg/m^3^ increase of NO_x_	Khreis et al. [10]
Hospitalizations, Respiratory disease	All ages	1.02 (1.01–1.03) per 10 μg/m^3^ increase of NO_2_	Héroux et al. [3]
Dementia	≥65 years	1.08 (1.03–1.13) per 5 μg/m^3^ increase of PM_2.5_	Yu et al. [11]

*<2500 grams

### Exposure assessment

Nitrogen dioxide (NO_2_) and particles with a diameter less than 2.5 µm (PM_2.5_) were measured outdoors at nine preschools in Malmö, during the spring of 2019 (3^rd^ to 7^th^ of April, *N* = 4 and 3rd of April to 10^th^ of June, *N* = 5) and again in the spring of 2020 (1^st^ of April to 1^st^ of June). The difference between the concentrations of air pollutants in 2019 and 2020 was estimated by calculating the average concentrations of NO_2_ and PM_2.5_ for each preschool in the spring of 2019 and then subtracting them with the corresponding values for the same period in 2020.

NO_2_ measurements were taken with the Swedish Environmental Institute’s diffusion samplers. These consist of a small cylinder that is open at both ends so that the air, including the ambient NO_2_, can flow through it. In the process, NO_2_ is absorbed in an impregnated filter in the cylinder. PM_2.5_ was measured with optical sensors (Purple Air PA-II-SD), which log PM_2.5_ concentrations continuously. These devices were placed under a rain cover at a height of about 2.0 m to be as close to the children’s ‘breathing zone’ as possible but also out of their reach. The differences were tested with Wilcoxon signed rank test.

### Outcome assessment

Calculations for the health outcomes of interest (mortality, preeclampsia, low birth weight, bronchitis, childhood asthma, hospital admissions for respiratory illness, and dementia) were performed according to their prevalence among different age groups. This baseline health data was derived from various sources, including the Swedish National Board of Health and Welfare, local health registers, and data from Statistics Sweden.

## Health impact assessment

A health impact assessment (HIA) based on air pollution exposure was performed using standard methods, described in more detail elsewhere [12]. The assumptions made regarding baseline population risk and the concentration-response functions (CRFs) utilized for this HIA are described in Table 1.

## Results

The average NO_2_ concentration in the spring of 2019 was 15.6 µg/m^3^ (range: 14.3–16.7 µg/m^3^) whereas the average NO_2_ level in the spring of 2020 had decreased to 9.0 µg/m^3^ (range: 8.3–9.6 µg/m^3^), the average difference being 6.6 µg/m^3^. The decrease ranged between 5.4 µg/m^3^ to 8.2 µg/m^3^, with a standard deviation of 3.8 µg/m^3^. The average PM_2.5_ level in the spring of 2019 was 8.3 µg/m^3^ (range: 5.4–13.0 µg/m^3^) and 6.5 µg/m^3^ in 2020 (range: 4.4–9.2 µg/m^3^), corresponding to a 1.9 µg/m^3^ decrease on average (ranging from an increase of 2.2 µg/m^3^ to a decrease of 5.0 µg/m^3^, standard deviation of 2.0 µg/m^3^). These results indicate a 42% decrease in NO_2_ concentrations (p-value = 0.004 from Wilcoxon signed rank test) and a 22% decrease in PM_2.5_ concentrations (p-value = 0.04 from Wilcoxon signed rank test) between 2019 and 2020.

The health impact assessment illustrated a substantial potential for a reduction in both mortality and morbidity, if the improved air quality would persist. For instance, assuming a permanent reduction of 6.6 µg/m^3^ in annual mean NO_2_ concentrations, would results in 25–39 fewer deaths each year in Malmö (Table 2), depending on the choice of CRF. If, instead of using the CRF from Stockfelt et al. [5], based on nitrogen oxides (NO_x_), the estimated number of deaths are much lower. An annual mean reduction in concentrations of PM_2.5_ of 1.9 µg/m^3^ would results in a prevention of 17 deaths per year when using a weighted CRF considering local, regional, and background sources (Table 2). However, applying a CRF based solely on local sources resulted in an estimated 69 preventable deaths.

**Table 2.** Estimated health effects (relative risk, RR) with a 95% confidence interval (95% CI) of improvement in air quality corresponding to the change in NO_x_, NO_2_ or PM_2.5_ between spring 2020 compared with spring 2019.

Health outcome	RR (95% CI)	Number of fewer outcomes per year (% of total number of cases)
Mortality		
	1.06 (1.03–1.08) per 10 μg/m^3^ increase of NO_2_	25 (1)
	1.08 (1.01–1.14) per 10 μg/m^3^ increase of NO_2_	39 (2)
	1.02 (1.01–1.04) per 10 μg/m^3^ increase of NO_x_	9 (0.3)
	1.06 (1.04–1.08) per 10 μg/m^3^ total increase of PM_2.5_	17 (1)
	1.26 (1.19–1.34) per 10 μg/m^3^ increase of local PM_2.5_	69 (3)
Morbidity		
Preeclampsia	1.13 (1.07–1.19) per 10 μg/m^3^ increase of NO_x_	3 (2)
Low birth weight*	1.09 (1.03–1.15) per 10 μg/m^3^ increase of PM_2.5_	7 (6)
Bronchitis	1.02 (0.99–1.06) per 1 μg/m^3^ increase of NO_2_	4 (4)
Asthma incidence	1.05 (1.02–1.07) per 4 μg/m^3^ increase of NO_x_	8 (2)
Hospitalizations, respiratory disease	1.02 (1.01–1.03) per 10 μg/m^3^ increase of NO_2_	11 (0.3)
Dementia	1.08 (1.03–1.13) per 5 μg/m^3^ increase of PM_2.5_	219 (11)

*< 2500 g.

The results furthermore suggest an annual decrease in cases of preeclampsia by 2%, children with low birth weight by 6%, children with bronchitis by 4%, cases of asthma by 2%, hospital admissions for respiratory diagnoses by 0.2%, and new cases of dementia by 11% if the improved air quality would have been permanent (Table 2).

## Discussion and implications

Several studies have shown that mobility patterns changed dramatically during the COVID-19 pandemic. The studies relied on different levels of lockdowns, an unsustainable path to a healthy urban environment. In Sweden, the pandemic response never included a lockdown, but those that could work from home were encouraged to do so, which was adopted in most office settings. This allowed for a real-world example of changed urban mobility and commuting patterns. Our findings illustrate that the reduction in air pollution levels in Malmö early in the pandemic was substantial and, if maintained, would entail considerable health gains. Mortality was estimated to potentially decrease by as much as 3% among Malmö’s population, again, if the observed improvements in air quality would continue long term. The potential to improve overall health was also evident, with, for example, a 2% and 11% reduction in new asthma and new dementia cases, respectively. While the measures taken during the COVID-19 pandemic are not sustainable long term, and the observed improvement in air quality may partly be due to other factors, the results indicate the potential for mitigating the adverse health effects of air pollution through large-scale behaviour change.

In line with our findings, a study evaluating air pollution concentrations from 1100 measurement stations across Europe reported that NO_2_ concentrations decreased in 2020 compared to the average concentrations recording during the same period of the previous 7 years at most locations [13]. Reductions in emissions from the transportation sector seemed largely responsible for the change. The health impacts of reduced NO_2_, PM_2.5_ and particles with a diameter less than 10 µm (PM_10_) concentrations in connection to COVID-19 related restrictions and lockdowns have also been estimated in France [14] and Italy [15], where cases of child asthma, hospitalization for respiratory diseases, risk of low birth weight, and mortality and lung cancer would decrease. While the present study did not investigate ozone (O_3_), evidence on how the restrictions related to the COVID-19 pandemic were seen to affect ozone concentrations across Europe is somewhat mixed. On one hand, the lockdown period highlighted that anticipated future reductions in NO_2_ levels in European urban areas may result in widespread increases in urban O_3_ pollution unless additional mitigation measures are implemented [16]. On the other hand, it has been suggested that a significant portion of the changes in O_3_ levels can be attributed to meteorological influences [17]. However, the limited data available from our study area (Malmö, Sweden) indicate a decrease in ozone rather than an increase [18].

Unlike most other studies on air quality reductions related to pandemic responses, it is important to note that Sweden never implemented a formal lockdown. Instead, among other recommendations, those that could work from home were encouraged to do so, which was adopted in most office settings. While this contributed to reduced mobility overall, 60% of Swedes still needing to commute to work switched from using public transportation to private cars [19]. The long-term impact of the transport sector on air pollution levels will vary depending on how these new patterns of mode of transport persist in the years following COVID-19. Overall, these altered mobility patterns should provide unprecedented incentive for the prioritization of active transport. The pandemic restrictions have furthermore introduced, catalysed, and normalized the prominence of virtual meetings and teleworking in many businesses and agencies. Such a transition has numerous advantages, including decreased business travel costs, reduced environmental impact, more efficient operations, saved time, and the increased opportunity for national and international cooperation. The present study indicates that these benefits could also extend to local air quality and, subsequently, public health. However, emissions are increasing again [20], meaning that the potential health gains from improved air quality suggested by the present study’s results are hypothetical.

## Methodological considerations

While we noted a decrease in air pollution levels from 2019 to 2020, it is essential to acknowledge that factors beyond pandemic-related mobility/commuting patterns may have contributed to this observed decline. However, there was a corresponding decrease in traffic flow from March to May 2020 in Sweden, with total road traffic volume dropping by more than 20% [21]. Additionally, the impacts of COVID-19 restrictions and lockdowns across Europe were reflected in lower NO_2_ concentrations in many cities [22], which also contributed to lower concentrations of long-range transported air pollutants in Sweden, positively impacting air quality [21]. Another factor to consider is that PM_2.5_ levels are heavily influenced by meteorological conditions, and variations observed between different years can be predominantly attributed to weather patterns. The Spring 2020 was considered a quite normal meteorological season albeit a bit dryer than normal [23]. It is possible, however, to isolate altered emissions due to other factors from expected changes due to meteorological factors using various methods. For instance, machine learning and statistical models exist to subtract predicted concentrations, adjusted for weather and time, from observed data using machine learning or statistical models with daily or hourly data from prior years [16,24]. In the present study, we chose not to undertake such calculations since our primary aim was not to establish causality between the observed differences in air quality and the pandemic-related restrictions but, rather, to underscore the potential significant health benefits associated with such observed differences following the natural experiment pandemic-related altered mobility patterns. Similarly, PM sensors can be sensitive to humidity, a potential source of measurement bias differences and the pandemic, but underscore the potential significant health benefits associated with such observed variations. Additionally, the measurement campaigns at Malmö preschools also included data from autumn 2018, which we opted to not include for two primary reasons, seasonality issues and notably higher levels in 2018. To ensure a more conservative approach and avoid exaggerating potential health benefits, we decided to use the 2019 spring data. The observed differences observed in the present study, based on a quite limited number of measuring locations (*N* = 9), may furthermore not be representative for the whole population in Malmö. It is also important to mention the potential for measuring error and other potential biases such as variations in light scattering by different particle types. Given that Malmö has consistent pollution sources (primarily traffic and long-range in transported PM) both spatially and temporally, and since our focus is on concentration differences rather than absolute values, the scattering effect is negligible. Improved air quality was also observed in Malmö city monitoring stations for 2020 ‘Luften i Malmö 2021’ report by the Malmö City Environmental Department [23]. Our decision to utilize data from our measuring campaigns instead of the monitoring stations by Malmö City Environmental Department was influenced by their broader geographical distribution across Malmö compared to the monitoring stations.

The discrepancy in health impact assessment outcomes for mortality estimates highlights the uncertainty surrounding the choice of CRF. The CRF from a cohort study in Copenhagen, Denmark [4], might be considered relevant in this study setting due to the proximity of Malmö to Copenhagen. Given the current circumstances where the observed decrease was likely to a large extent due to reductions in local emissions, however, we anticipate that the higher CRF would be more suitable in this scenario [25]. It is furthermore important to note that the present study specifically focused on the health effects resulting from improved air quality and did not include potential impacts from other pandemic-related mobility and behavioural changes. Moreover, the chosen health outcomes align with current epidemiological knowledge, but this is a continuously developing field.

## Conclusion

Decreases in air pollution concentrations were observed in Malmö, Sweden, during the early stage of the COVID-19 pandemic when commuting and mobility patterns changed. If permanent, these reductions would contribute to prevented deaths and a wide range of adverse health outcomes in the study population. While the state of pandemic restrictions is not sustainable, and the reductions observed may partly be due to other factors, these findings indicate the potential for reducing air pollution levels through behaviour change, such as through altered transportation and mobility patterns.
